# *QuickStats:* Emergency Department Visit Rates* Related to Mental Health Disorders,^†^ by Age Group and Sex — National Hospital Ambulatory Medical Care Survey, United States,^§^ 2016–2018

**DOI:** 10.15585/mmwr.mm6948a13

**Published:** 2020-12-04

**Figure:**
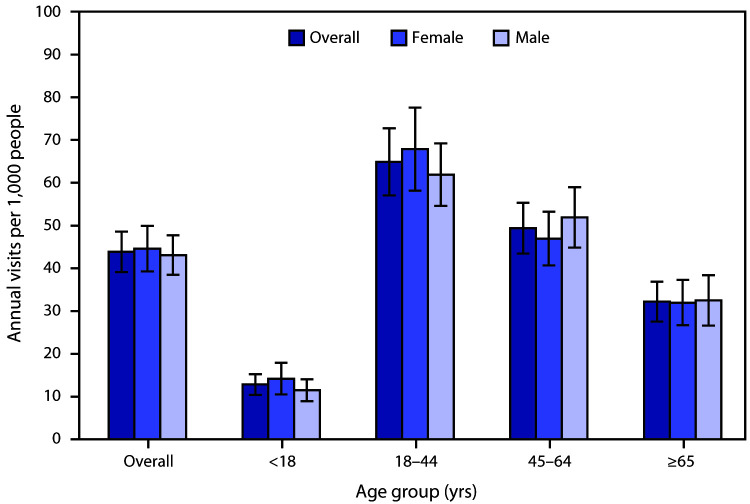
During 2016–2018, there were 43.9 emergency department visits per 1,000 persons per year with a diagnosis of a mental health disorder. Rates were lowest among children and adolescents aged <18 years (12.8) and highest for adults aged 18–44 years (64.9). Rates declined with age for adults aged 18–44 to ≥65 years (32.2). Overall and for each age group, there were no statistically significant differences by sex.

**Source:** National Center for Health Statistics. National Hospital Ambulatory Medical Care Survey, 2016–2018. https://www.cdc.gov/nchs/ahcd/ahcd_questionnaires.htm.

